# Death of Lisfranc

**Published:** 1847-07

**Authors:** 


### DEATH OF LISFRANC.

Our Paris correspondent mentions the death of the eminent surgeon Lisfranc, which occurred on the 9th of May. Lisfranc was surgeon to *La Pitié* hospital, and one of the most distinguished members of the profession in France.

